# Cosmochemical fractionation by collisional erosion during the Earth's accretion

**DOI:** 10.1038/ncomms9295

**Published:** 2015-09-23

**Authors:** Asmaa Boujibar, Denis Andrault, Nathalie Bolfan-Casanova, Mohamed Ali Bouhifd, Julien Monteux

**Affiliations:** 1 Laboratoire Magmas et Volcans, Université Blaise Pascal, CNRS UMR-6524, 5 rue Kessler, 63000 Clermont-Ferrand, France

## Abstract

Early in the Solar System's history, energetic collisions of differentiated bodies affected the final composition of the terrestrial planets through partial destruction. Enstatite chondrites (EC) are the best candidates to represent the primordial terrestrial precursors as they present the most similar isotopic compositions to Earth. Here we report that collisional erosion of >15% of the early Earth's mass can reconcile the remaining compositional differences between EC and the Earth. We base our demonstration on experimental melting of an EC composition at pressures between 1 bar and 25 GPa. At low pressures, the first silicate melts are highly enriched in incompatible elements Si, Al and Na, and depleted in Mg. Loss of proto-crusts through impacts raises the Earth's Mg/Si ratio to its present value. To match all major element compositions, our model implies preferential loss of volatile lithophile elements and re-condensation of refractory lithophile elements after the impacts.

## Abstract (web summary)

Collisions in the early Solar System affected the final composition of the terrestrial planets, and enstatite chondrites (EC) are thought to represent the primordial Earth's precursors. Here, the authors show that differences between Earth and EC are due to impact erosion of >15% of the early Earth's mass.

## Graphical abstract

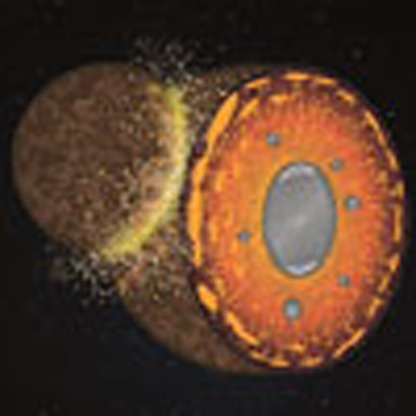

Large-scale melting occurred broadly in the first stages of planetary accretion^1^. The impressive homogeneity of stable isotopic compositions of large bodies suggests extensive melting and formation of magma oceans^1^. However, while large-scale melting can efficiently erase isotopic heterogeneities, low degrees of partial melting are a primary cause of chemical segregation. The possibility to form proto-crusts by low degrees of melting of chondritic material is evidenced by the discovery of felsic achondrites (GRA-06128/9; ref. 2). Early differentiation of planetary embryos was also recently suggested by a study that determined the initial content of the short-lived radionuclide ^26^Al in angrites^3^. Therefore, early crusts could form by different processes, such as fractional crystallization of a magma ocean or the migration of silicate melts over networks of veins and dikes^4^. Despite the formation mechanism of these proto-crusts, their occurrence on accreting bodies should have played a major role in the final planetary composition, because energetic episodes of accretion have eroded the planetary surfaces^5^,^6^,^7^,^8^.

The chemical composition of the building blocks that accreted to form the Earth remains controversial. Our planet shows remarkable isotopic similarities with enstatite chondrites (EC), especially with those of the EH type^9^, for the elements whose isotopes do not fractionate during core segregation (O, Ca, N, Mo, Ru, Os, Cr, Ni, Ti and Sr). However, EC and the Earth present important chemical differences. First, EC are so reduced that their silicate phases are free of FeO, which differs from the present-day silicate mantle with 8 wt% FeO. This issue may eventually be solved by internal oxidation processes for some of the Fe metal^9^,^10^. A second issue is that EC show a Mg/Si weight ratio lower than 1 (∼0.63; ref. 11), differing substantially from that of the Earth's upper mantle (∼1.1; ref. 12). The Mg/Si ratio of bulk Earth (BE) could be slightly overestimated if the lowermost mantle is bridgmanitic (that is, perovskitic) rather than pyrolitic^13^. Furthermore, the Earth shows higher abundances of refractory lithophile elements (RLE) and lower concentrations of moderately volatile elements (as the alkali elements Li, Na, K and Rb) compared with EC and all other chondrites (Fig. 1).

In this study, we test whether collisional erosion of early crusts can explain the chemical divergence between the BE and EC. Based on experiments on the melting properties of synthetic EC at pressures between 1 bar and 25 GPa, we show that early differentiated planetary bodies can develop silica- and alkali-enriched crusts. Loss of these crusts through impact erosion can ultimately increase the Mg/Si ratio of the planetary bodies to match the current Earth ratio. In addition, to further increase the budget in RLE and accurately reproduce the terrestrial concentrations of the major and minor elements, impact erosion must be accompanied with preferential loss of volatile lithophile elements and re-condensation of RLE.

## Results

### Partial melting of enstatite chondrites

We experimentally investigated the composition of melts produced by low degrees of melting of synthetic EC powders, at low oxygen fugacity (3.6 to 1.8 log units below the iron/wustite buffer), at different pressure conditions expected for melt segregation in partially molten planetary embryos (see Methods section and Supplementary Fig. 1). Our pseudo-eutectic melts are all characterized by high concentrations of SiO_2_, Al_2_O_3_ and Na_2_O, and low MgO contents (Fig. 2). The change with pressure of the low-degree melt composition agrees with that previously reported at 1 bar (ref. 14). The most striking features are the increase of MgO (Fig. 2b) and decrease of SiO_2_ (Fig. 2a) and Al_2_O_3_ (Fig. 2c) with pressure. The disappearance of clinopyroxene at 16 GPa and garnet at 24 GPa (ref. 15) induces major CaO (Fig. 2d), Na_2_O (Fig. 2e) and K_2_O (Fig. 2f) enrichment of the liquid, respectively. Altogether, pseudo-eutectic liquids show compositions between rhyolitic and trachy-andesitic in the range of pressures investigated. In partially molten planetesimals, such melts should ascend relatively easily towards the planetary surface due to their low melt densities, even for low degrees of partial melting^4^. As the melts can re-equilibrate during their ascent to the surface, those produced at shallower depths are more likely to stay unaltered and produce proto-crusts enriched in incompatible elements. The most appropriate melts for the formation of proto-crusts should then be those produced at relatively low pressures and degrees of partial melting.

### Change of proto-Earth composition with collisional erosion

We now evaluate how collisional erosion of proto-crusts made of these pseudo-eutectic liquids would affect the chemical composition of an EH-type planetary embryo^11^. First, we calculate the Mg/Si ratio of a planetary body after removal of pseudo-eutectic melts generated at average pressures of 1 bar to 25 GPa and compare it with the BE^12^, with the hypothesis that ∼7 wt% Si is present in the Earth's core^9^,^16^. By increasing the amount of crustal erosion, the Mg/Si ratio of the depleted EH planetoid increases towards the present-day BE ratio of ∼0.9, owing to the high SiO_2_ content of the melts (Fig. 3a). The lower the pressure of melt-solid equilibration, the higher the SiO_2_ content in the melt, and therefore the crustal erosion should be the less extensive. The BE Mg/Si ratio can be achieved by accretion of EH chondrites and erosion of a crust of 15–18% of the planetary mass for solid-melt equilibrium at pressures below 10 GPa. This amount of crustal erosion is comparable to the loss of highly incompatible elements required to explain the mantle budget in volatile elements^17^, and is comparable to the amount of mass lost during hit and run simulations^18^. As the formation of such SiO_2_-rich crust necessitates a low degree of partial melting (at a level of 5–7%), removing proto-crust to a level of 15–18 wt% of the planetary mass requires repeated processes of proto-crust formation and collisional erosion. Repeated partial melting of the EH-type mantle would constantly produce an SiO_2_-rich proto-crust, owing to the fixed pseudo-eutectic composition. The nature of planetary accretion itself provides the necessary energy to melt planetesimals and erode them or even disrupt them (see below). Simulations indicate that an accreting proto-planet should experience ∼10^5^ collisions^18^.

Removal of proto-crust also affects the Al/Si, Ca/Si and Na/Si ratios (Fig. 3b and Supplementary Fig. 2). Interestingly, the misfits between Mg/Si, Al/Si, Ca/Si and Na/Si ratios in BE and in our model of EH-type planetoid depleted by crustal erosion show a clear correlation with the condensation temperatures of the elements of interest^19^ (Fig. 3b). Rather than being fortuitous, this trend can be understood in terms of differential re-condensation of the elements after collisional erosion. Our model implies that early differentiation of a planetary embryo forms a silica-rich crust (Fig. 4a,b) that is subsequently eroded and vaporized by energetic impacts (Fig. 4c). The eroded material is then chemically fractionated with a preferential condensation of the refractory elements relative to the volatiles (Fig. 4d). This explains the previously reported marked consequences on the thermal history of Earth that could result from the loss of incompatible and refractory heat-producing elements, such as U and Th^5^,^20^,^21^.

In fact, the Earth's budget in RLE can be reached by a number of different erosion and re-condensation models. The lowest amount of erosion (15–18 wt% of proto-crust to meet the right Mg/Si ratio) requires re-condensation of 100% of the refractory elements (Ca and Al) on the planetary surface (Fig. 3c). For higher amounts of planetary surface erosion (15–18 wt% proto-crust plus a mantle fraction), the models require less chemical fractionation during vaporization or re-condensation. For 40% mantle erosion, re-condensation of 84% of Al, 73% of Ca, 10% of Mg and 5% of Si is necessary (Fig. 3c and Table 1) to produce a planetary composition similar to Earth. Comparable fractionation of major elements with Ca- and Al-rich condensates depleted in Mg and Si was reported in the most primitive unequilibrated Semarkona ordinary chondrites (LL3.0; ref. 22). Also, such a degree of chemical fractionation during re-condensation may not be necessary if the composition of the parent body deviates from the EH chondrites (see below). Then, non-equilibrium processes, such as solar wind, could have favoured the loss of volatile elements from the gravitational field of the proto-planet. In addition, the volatile elements could have been partially atmophile due to hot planetary surfaces, which could have contributed to the volatile and refractory fractionation.

### The major sources of energy provided by planetary accretion

During planetesimal growth, the thermal state of a proto-planet depends on its initial heating caused by the decay of short-lived radionuclides such as ^26^Al and ^60^Fe (ref. 23), on its accretionary history and on how potential energy is dissipated during the iron/silicates segregation. Temperatures in excess of 2,000 K could have been reached within the first 2–3 million years after the formation of the first solids of the Solar System (calcium–aluminium-rich inclusions, CAI)^24^. The early radioactive heating can therefore cause both segregation of a metal-rich core and silicate melting on planetary embryos that have quickly accreted (to attain a radius of 30 km within the first 3 million years; see Fig. 2 in Yoshino *et al*.^24^). Kleine *et al*.^25^ showed that high temperatures of early asteroids are in agreement with the young ages of iron meteorites, OC, CO and CR chondrites and the peak temperatures recorded by the same meteorites (see in ref. 25).

In addition to this radioactive heating, in the shallow parts of the impacted planet, impact heating (Δ*T*), superimposed to a sufficiently hot proto-planetary interior (with an initial temperature of *T*_0_) can lead to temperatures (*T*=*T*_0_+Δ*T*) much larger than the vaporization temperature of silicates (∼1,300 °C at 0.001 bar; see Fig. 5 above). This heating is localized in a spherical region called the isobaric core, just beneath the impact site (for example, ref. 26). By making the conservative assumptions that (i) kinetic energy of the impactor is controlled by the escape velocity of the impacted body, (ii) impactor and target body have the same densities and (iii) only 30% of the incoming kinetic energy is converted into heat, a simple energy balance indicates that Δ*T* (K)=4.7 × 10^−5^ × *R*^2^ (km) (refs 26, 27).

Assuming that the impact occurs with the escape velocity of the impacted body is a conservative assumption in the sense that it is the minimum impact velocity; larger impact velocities can substantially increase the post-impact temperature below the surface. For instance, some episodes of hypervelocity impacts have probably played a major role during the accretion of the terrestrial planets. For example, a recent model suggests that Jupiter and Saturn have likely migrated inwards and then outwards early in the Solar System history^28^, leading to strong resonance and high eccentricities of the planetesimals. As the resonance with Jupiter can significantly increase the impact velocities^29^, the kinetic energy, available during the impact processes on growing bodies, may have enhanced the vaporization and the excavation of their shallow material. This model, with marked migration of Jupiter and Saturn, is one suggested model of the dynamical evolution of the Solar System. Other models may not produce such high velocity collisions, but they would have a longer period during which frequent collisions could occur.

Vaporization following collisional erosion from large impacts is widely accepted^30^. The abundance of impacts is supported by observations of the iron meteorites, which represent fragments of cores of differentiated bodies, which are now destroyed. These iron meteorite parent bodies are as old as the CAI, which are the oldest objects of the Solar System, that formed during the first million year after *T*_0_ (ref. 31). Thus, collisional stripping operated in the very early stages of the formation of the Solar System.

## Discussion

Our experimental results show that surface erosion modifies more efficiently the planetary composition of smaller planetary bodies owing to lower internal pressures (Fig. 3a). This suggests a more efficient chemical fractionation early in the accretion history. As a matter of fact, silica- and alkali-rich compositions (andesitic to rhyolitic and trachyitic) have been observed in various asteroidal materials: bulk achondrites (GRA-06128/09), silicate enclaves in iron meteorites, glass inclusions or igneous clasts in aubrites, glass inclusions in the howardite, eucrite and diogenite meteorites, clasts in the ureilites. The oldest sampled materials of the Solar System with a granitic composition are only 5–30 Myr younger than the formation of the Solar System (see ref. 32 for a review). This indicates the formation of silicic crust nuclei very early in the inner Solar System.

A widespread Si isotopic dichotomy is observed between planetary/asteroidal bodies and the chondrites. There is a correlation between the enrichment in heavy Si (^30^Si) with both (i) the depletion of the moderately volatile elements (K) and (ii) the depletion of Si relative to Mg for the Earth, the Moon, Vesta, Mars, the angrites (basaltic meteorites) parent body and the chondrites^33^. As suggested by Pringle *et al*.^33^, this correlation could be caused by early impact-induced evaporative loss, occurring as early as the formation of angrites, 2 Myr later than the formation of the oldest currently known objects of the Solar System^34^,^35^ (Supplementary Note 1). Thus, the Si isotopic measurements fully support our model of early collisional erosion. It has also been proposed that energetic episodes of impacts can induce significant chemical fractionation of RLE in EL chondrites^36^.

**Figure 1:**
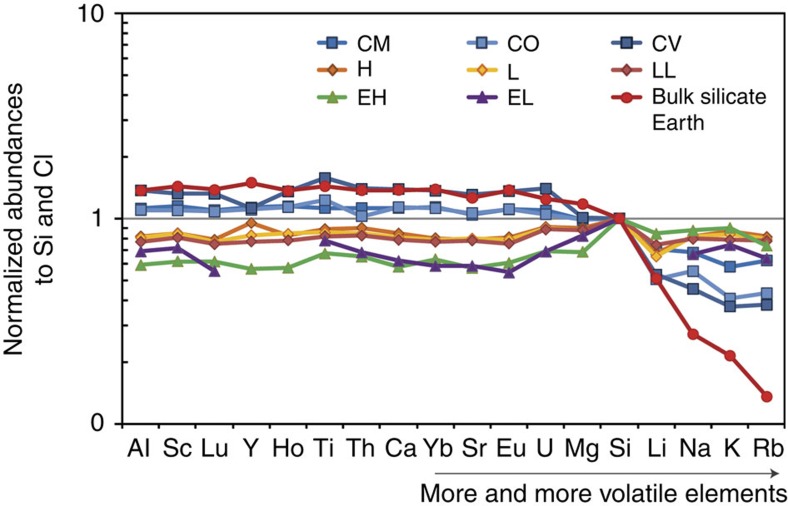
Major chemical differences between the chondrites and the bulk silicate Earth (BSE). The abundances are normalized to Si and CI chondrites^11^,^12^. From left to right, the lithophile elements are reported with decreasing their 50% condensation temperature. The depletion of volatile elements in the BSE results from the erosion of crusts enriched with incompatible elements and the subsequent loss of the most volatile elements.

**Figure 2:**
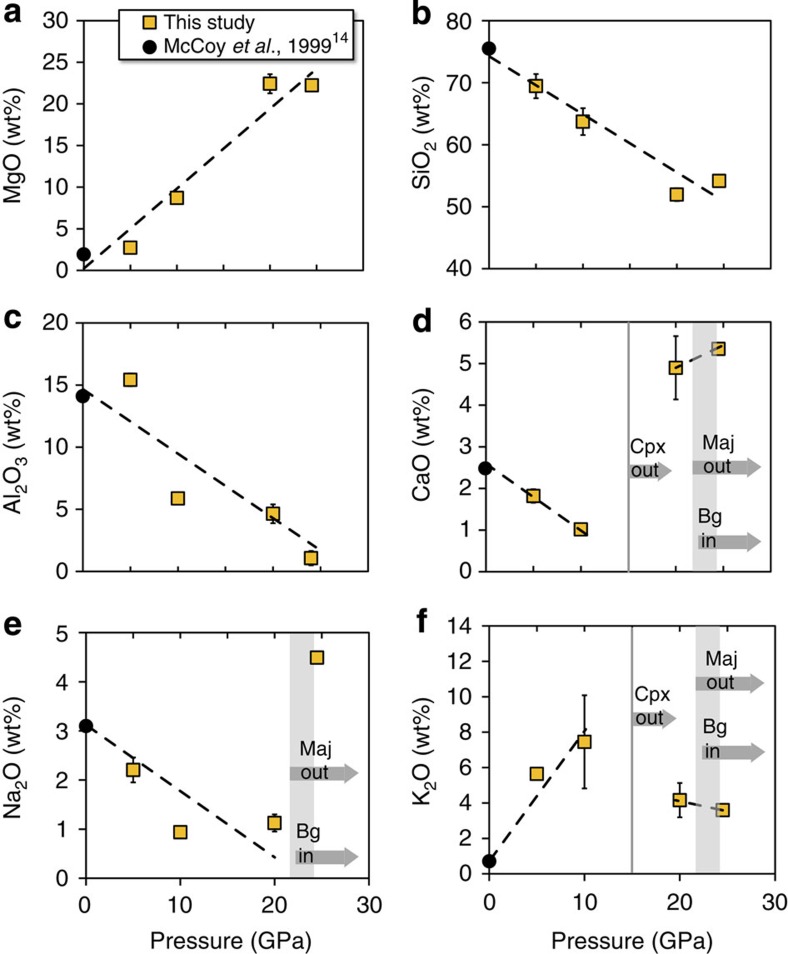
Chemical composition of pseudo-eutectic melts synthesized at high pressures. We report the compositions of melts generated by partial melting of an EC-like mantle at pressures between 5 and 25 GPa (yellow squares). Our data set plots are in agreement with previous results performed at 1 bar (ref. 14) (dark circles). Whereas MgO content (**a**) increases with pressure in the silicate melts, the concentrations of SiO_2_ (**b**) and Al_2_O_3_ (**c**) decrease. Sharp changes of the liquid compositions occur when the sample encounters phase transformations (shown with the grey arrows), in particular, with the disappearance of clinopyroxene (Cpx) at 15 GPa (vertical lines in **d** and **f**) and simultaneous disappearance of majorite (Maj) and appearance of bridgmanite (Bg, the MgSiO_3_ perovskite; vertical grey areas in **d**–**f**) at 24 GPa.

In contrast to the other planetary differentiation processes, such as core segregation or magma ocean crystallization, an early impact erosion affects the composition of both planetary bodies and the remaining unaccreted material, in a complementary way. Owing to a higher surface/volume ratio compared with the planetesimals, the chondritic material that was left over from the accretion may have preferentially reacted with gases produced by vaporization of the eroded crusts, which are enriched in SiO_2_ and volatile lithophile elements. This can explain the enrichments in Na, S and Si observed at the edges of chondrules contained in carbonaceous and ordinary chondrites^37^,^38^,^39^. EC are the most alkali- and silica-rich chondrites^11^,^40^ (Fig. 1), but in contrast to the carbonaceous chondrites, this enrichment is global and not limited to chondrules (Supplementary Note 2). This is likely owing to the fact that EC have experienced important thermal metamorphism that usually erases the chemical gradients. Several studies have analysed the behaviour of alkali elements in chondrules in order to retrieve the conditions under which the chondrules form. The majority of these studies concluded that very high dust densities with high solid/gas ratios are required in the nebula to enrich the edges of chondrules in Na^37^. This was previously attributed to aqueous alteration^41^. In light of our results, we propose that the destruction and vaporization of eroded proto-crusts can also contribute to this elevation of the dust/gas ratio of the nebula in the formation regions of chondrules.

**Figure 3:**
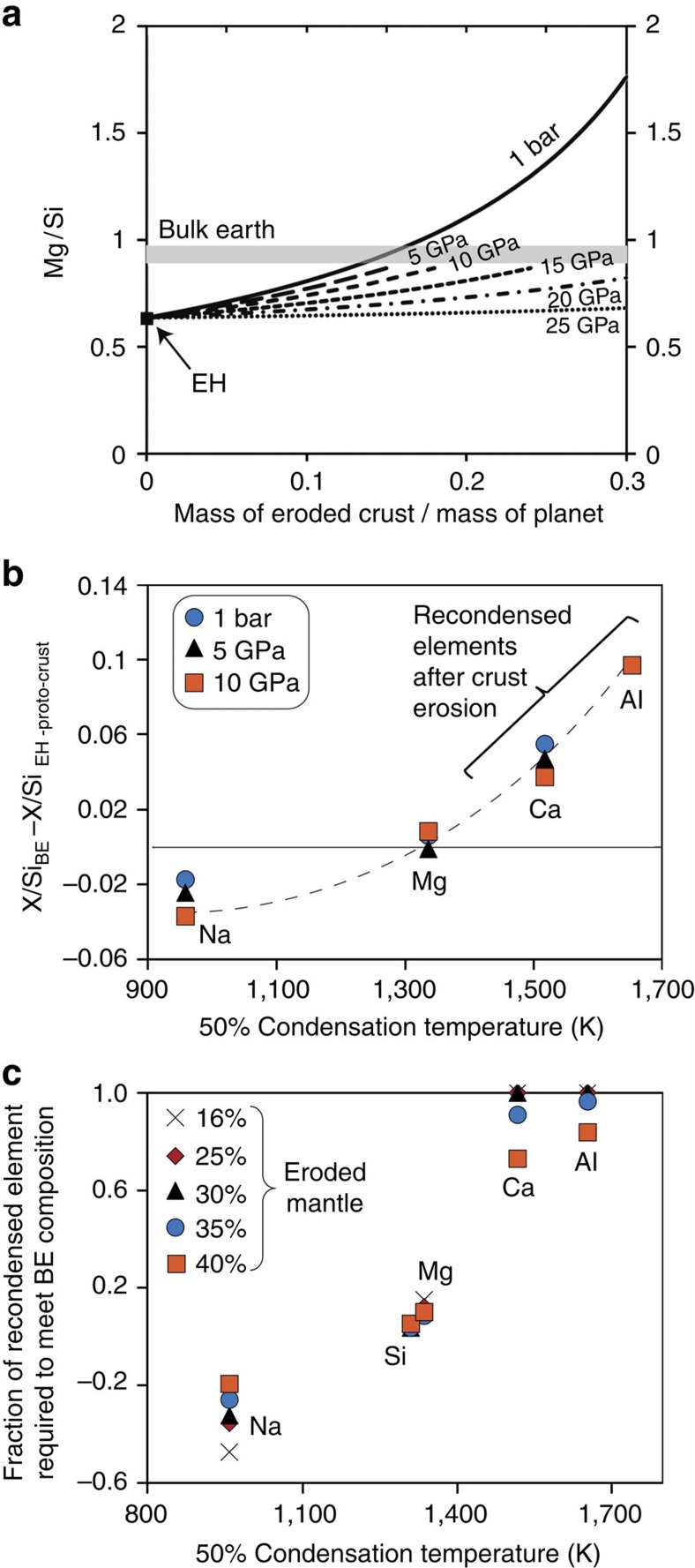
Change by collisional erosion of the proto-planetary chemical composition. (**a**) Change by collisional erosion of the planetary Mg/Si ratio by removal of a differentiated crust with a composition equivalent to that of pseudo-eutectic melts produced at pressures of 1 bar and 5, 10, 15, 20 and 25 GPa. Removal by collisional erosion from the model EH composition results in an increase of the Mg/Si ratio of the planetesimal. The grey area represents the Mg/Si ratio of the BE with 7 wt% Si dissolved into the core^9^,^16^. Erosion of a crust of >15% of the planet mass is required to reconcile the Mg/Si ratio of an EH-type planet with the present-day BE^12^. (**b**) Correlation between residual misfits between BE and the EH proto-planet X/Si for X=Na, Ca and Al as a function of the 50% condensation temperature of the elements^19^. After the mass of eroded crust is adjusted to meet the Mg/Si ratios of BSE, there is a residual misfit for the abundances of other major elements (Supplementary Fig. 2). The correlation between BE enrichments and the condensation temperatures of the different elements suggests chemical fractionation during the processes of vaporization of the planetary surface, with re-condensation of the eroded material on the planetary surface (Fig. 4). (**c**) Degree of chemical fractionation required by our model, for different amounts of collisional erosion. Here we consider that the 15% of the crust (produced in the 0–5-GPa range) required to match the Mg/Si ratio of the BE and 16–40% of the planetary mantle are eroded by the impacts. For a total erosion of 31–45% of the planetary mass, for example, the actual Na/Si, Mg/Si, Ca/Si and Al/Si ratios of BE are reproduced when 100% of Al and Ca, 10% of Mg and 5% of Si are re-condensed on the planetary surface, which is in agreement with their condensation temperature. A negative value for Na denotes the fact that the residual mantle of our EH-type model (after collisional erosion) would still contain high Na contents compared with BE. In this case, additional Na volatilization from the residual mantle is required.

**Figure 4:**
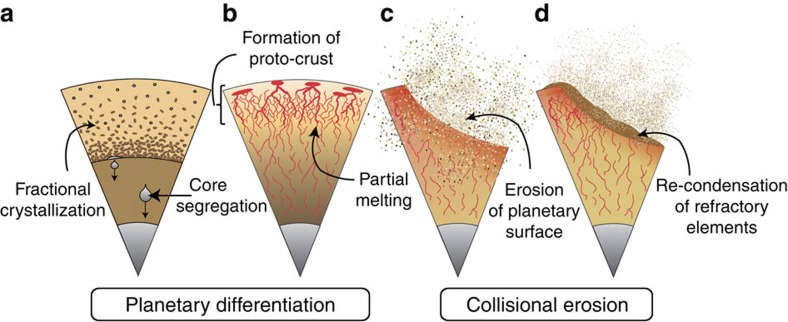
Schematic model of chemical fractionation by collisional erosion. (**a**,**b**) Early heating leads to the rapid segregation of Fe-rich metal into the core. It is accompanied with the formation of a deep magma ocean^1^ (**a**) and/or small-scale partial melting and formation of complex networks of veins and dikes^4^ (**b**) which allows fast transfer of melts to the surface^4^. Both can yield compositional stratification of the mantle and the formation of an SiO_2_-rich proto-crust. (**c**) The repeated collisions induce erosion of the proto-crust enriched in incompatible elements, as well as part of the planetary mantle. (**d**) Within the fraction of material volatilized by meteoritic impacts, re-condensation of refractory elements is favoured compared with the volatile elements.

**Figure 5:**
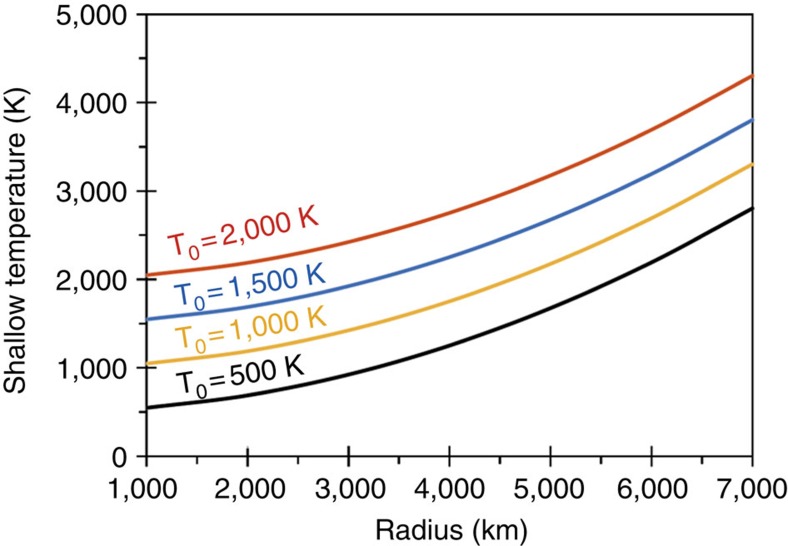
Shallow post-impact temperature as a function of the target radius R. Different pre-impact temperatures are illustrated (from *T*_0_=500 to 2,000 K). We consider that (i) surface gravity is equal to *g*=4/3 π G ρ R (G is the gravitational constant and ρ is the density of the target), (ii) kinetic energy of the impactor is controlled by the escape velocity of the impacted body (that is, *v*_impactor_=√(2*gR*)), (iii) the impactor and impacted body have the same densities, and (iv) only 30% of the incoming kinetic energy is converted into heat.

The EH meteorites that are sampled today could have suffered an SiO_2_ enrichment compared with their parent bodies, owing to interaction with crusts eroded from primordial planetesimals. This would have drifted their composition away from that of the Earth, compared with the original building blocks (Fig. 1). This common original material could have had a higher Mg/Si ratio and lower content in alkali elements than the EH chondrites. Consequently, our quantitative model of collisional erosion developed in this study (for example, Fig. 3) can be considered as the most extreme one, as less proto-crust erosion would be required to meet the actual BE chemical composition. Altogether, we show that collisional erosion could readily explain the major chemical divergences between the EH chondrites and Earth. It reinforces the model of the EH-like Earth^9^, nevertheless adding to this model the idea that the common starting material could have been slightly depleted in SiO_2_ and volatile lithophile elements in comparison with the EH chondrites.

## Methods

### Preparation of starting materials

The starting material was composed of 68 wt% silicate and 32 wt% metal with a chemical composition equivalent to the average composition of EC^9^ (Supplementary Table 1). While we are exclusively interested in the silicate properties, the presence of metals helps reproduce the EH-Earth conditions more precisely and buffer the oxygen fugacity. The silicate fraction was composed of ultra-pure oxides and carbonates (SiO_2_, MgO, Al_2_O_3_, TiO_2_, Cr_2_O_3_, MnO, Na_2_CO_3_, CaCO_3_ and K_2_CO_3_) that were finely ground together, decarbonized and then dried overnight at 1,000 °C. The metal powders were composed of a fine mixture of ultra-pure Fe, Ni, Si, Mn, Co and/or FeS. All samples were S free, except for 5 wt% S in the metal of sample no. 104. Silicate and metal fractions were intimately mixed to obtain a homogeneous chondritic powder, that was kept constantly in a vacuum oven to avoid hydration.

### Details of the experimental conditions

We conducted experiments at 5, 10, 20 and 25 GPa, with temperatures ranging from 1,380 to 1,900 °C using Kawai-type 1,000-t and 1,500-t multi-anvil presses. We heated the experiments to temperatures at or slightly above the solidus, that is, between 1,380 and 1,900 °C, for oxygen fugacities between −2.1 and −3.6 log units below the IW buffer (Supplementary Table 2). Assemblies were composed of Cr-doped MgO octahedra pressure media with edges of 18, 14 and 10 mm, coupled with tungsten carbide cubes with 11, 6 and 4 mm truncations, respectively. Pressure calibrations of both 1,000-t and 1,500-t presses were previously described^42^. The sample powder was loaded into a graphite capsule that was surrounded by MgO sleeves to prevent the sample pollution. High temperatures were achieved using an LaCrO_3_ furnace and a ZrO_2_ sleeve thermal insulator. Temperature was measured using a W_5_Re/W_26_Re thermocouple in experiment nos. 1,223 and 174, and estimated using the relation between temperature and electrical power for experiment nos. 1,216 and 104. Pressure and temperature uncertainties are estimated to be ∼0.5 GPa and 100 °C, respectively.

To achieve homogeneous samples without relict of the starting powders, we first heated the samples above the liquidus temperature^43^ for a couple of minutes. Temperature was then rapidly reduced (with a rate of 100 K in <20 s) to a temperature just above the reported solidus. The sample was then equilibrated between 30 min and 3 h. This procedure helps grain growth and allows segregation of relatively large pools of melt, which are usually difficult to collect with low degrees of partial melting^43^.

### Chemical compositions of the coexisting phases

The microstructure of recovered samples was observed using a scanning electron microscope (Supplementary Fig. 1). Phase relations and chemical compositions were determined using a CAMECA SX100 electron probe micro-analyser (Supplementary Table 1). We used an accelerating voltage of 15 kV, an electron beam defocused to 2–20 μm and a current of 15 nA, except for the chemical analyses of bridgmanites that were analysed with 2 nA. As standards, we used pure metals and silicates.

## Additional information

**How to cite this article:** Boujibar, A. *et al*. Cosmochemical fractionation by collisional erosion during the Earth's accretion. *Nat. Commun.* 6:8295 doi: 10.1038/ncomms9295 (2015).

## Supplementary Material

Supplementary Information Supplementary Figures 1-2, Supplementary Tables 1-2, Supplementary Notes 1-2 and Supplementary References

## Figures and Tables

**Table 1:** Resulting compositions with dual processes of collisional erosion and fractional re-condensation.

	**Bulk Earth**	**Assumed core composition**^44^	**EH-like planet**			**EH-like planet with adjusted core size**		
			**0% eroded mantle**	**25% eroded mantle**	**40% eroded mantle**	**0% eroded mantle**	**25% eroded mantle**	**40% eroded mantle**
Mg	15.6		13.8	12.8	10.8	15.9	15.8	15.6
Al	1.6		1.0	1.1	1.1	1.2	1.4	1.6
Si	16.6	7	15.0	13.7	11.5	17.2	16.9	16.6
Ca	1.7		1.1	1.2	1.2	1.3	1.5	1.7
Na	0.2		0.2	0.2	0.1	0.2	0.2	0.2
K	0.0		0.0	0.0	0.0	0.0	0.0	0.0
O	30.5	4	27.9	25.8	21.9	30.5	30.5	30.5
Fe	31.4	82	38.0	41.9	49.6	31.4	31.4	31.4
Co	0.1	0.1	0.1	0.1	0.1	0.1	0.1	0.1
Ni	1.6	5	2.3	2.5	3.0	1.6	1.6	1.6
S	0.6	2	0.6	0.6	0.7	0.6	0.6	0.6

We compare the actual bulk Earth composition (first column) (calculated based on bulk silicate Earth^12^ composition and cosmochemical estimates of core composition^44^ (second column)) with the EH-like Earth (third column) after erosion of (i) 15% of a crust composed of pseudo-eutectic melts produced at 0–5 GPa and (ii) 0–40% of its mantle. The erosion yields a significant depletion in the lithophile elements (Mg, Si, O, Na, Al and Ca) compared with the siderophile elements (Fe, Ni, Co and S). By adjusting the core size to the actual terrestrial core size (by fixing the concentrations of the siderophile elements to that of the Bulk Earth), the composition of our EH-like planetary model can reach values very close to that observed in the present-day Earth.
