# Supplementary material for: Cosmochemical fractionation by collisional erosion during the Earth's accretion
Source: Nat Commun. 2015 Sep 23;6:8295. doi: 10.1038/ncomms9295 (PMC4667431; doi:10.1038/ncomms9295)
Supplement: Supplementary Information — Supplementary Figures 1-2, Supplementary Tables 1-2, Supplementary Notes 1-2 and Supplementary References [file ncomms9295-s1.pdf]

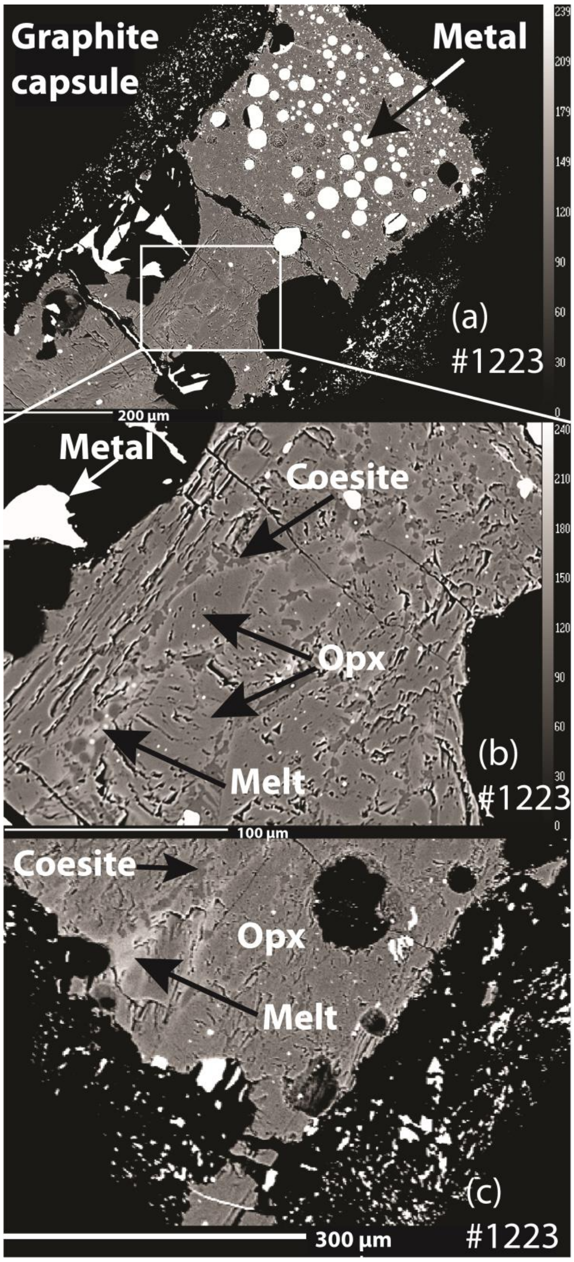

Supplementary Fig. 1: Texture of sample #1223 synthesized at 5 GPa and 1380°C. The microstructure is compatible with the presence of a small thermal gradient: a colder part is free of silicate melt and composed of metal blobs embedded between crystals of enstatite and coesite (frame (a)); a central zone is composed of larger grains of enstatite and coesite coexisting with small pockets of silicate melt at the grain boundaries (frame (b)); the hotter part of the sample exhibits the same texture than the central region but with a larger pool of silicate melt collected at the capsule extremity (frame (c)). Melt pockets located close to the un-molten regions have experienced near-solidus conditions. All melts analysed in this sample show comparable compositions, as the degree of partial melting remained limited, even in the hottest part of the sample.

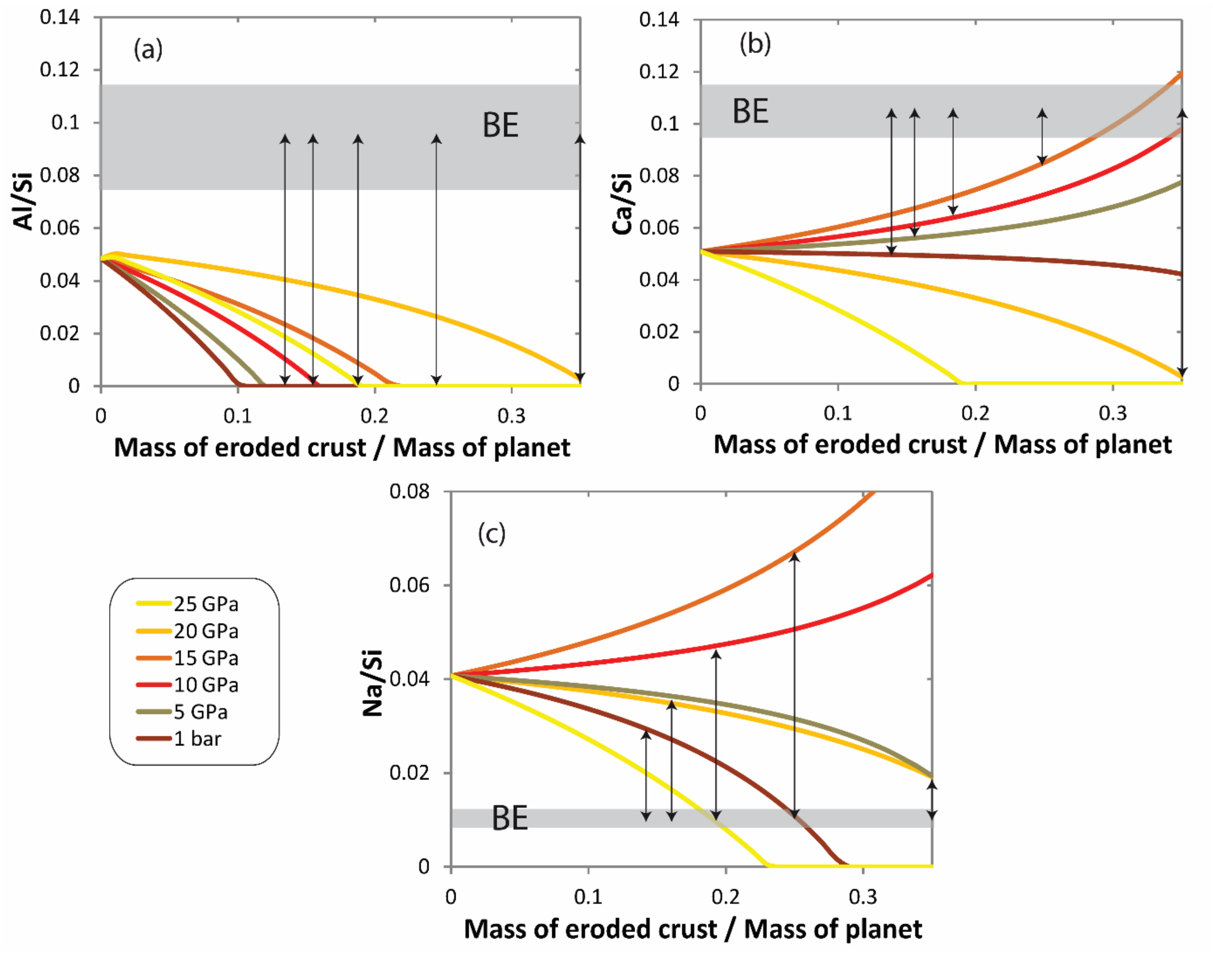

Supplementary Fig. 2: Effect of crust removal on major element ratio. Here we report the evolution of the bulk (a) Al/Si, (b) Ca/Si and (c) Na/Si ratios of an EH-type planet after (i) sequestration of 7 wt% Si in the core[1-3] and (ii) collisional erosion of crusts generated at pressures from 1 bar to 25 GPa (coloured curves). Vertical black arrows are the misfits (X/Si_{BE} - $X/Si_{EH\text{-}Protocrust}$) observed for Al/Si, Ca/Si and Na/Si ratios between the BE[4-7] and the crust-eroded EH-like planet, when Mg/Si ratios reaches the BE value of 0.9 (when the curves in Fig. 4a are crossing the grey area). In this calculation, we consider a proto-crust of fixed composition for each nominative average pressure of melt formation. The considered chemical composition of the melts at 1 bar is from McCoy et al. (1999)[8].

Supplementary Table 1: Chemical compositions of the starting compositions and the coexisting phases of samples (wt%).

Starting composition (68 wt% silicate, 32 wt% metal)												
Silicate	SiO_2	TiO_2	Al_2O_3	Cr_2O_3	FeO	MnO	MgO	CaO	Na_2O	K_2O		Tot
	62	0.21	2.6	0.19	0	0.04	32.2	1.3	1.4	0.14		
Metal	Si	Cr	Fe	Mn	Ni	Co	S					
Without S	2.5	0.68	90.4	0.91	5.32	0.23	0					
With S	1.5	0.65	86.7	0.88	5.1	0.22	5					
Silicate melts compositions												
	SiO_2	TiO_2	Al_2O_3	Cr_2O_3	FeO	MnO	MgO	CaO	Na_2O	K_2O	La_2O_3	Tot
#1223	69.45	0.66	15.42	0.19	0.7	0.4	2.73	1.82	2.21	5.64	0.09	99.3
N=4	±1.96	±0.08	±0.52	±0.06	±0.16	±0.08	±0.05	±0.16	±0.25	±0.21	±0.07	
#1216	61.18	3.42	5.64	0.37	6.14	0.26	8.43	1.96	0.88	7.34	1.61	96.21
N=7	±2.17	±0.15	±0.27	±0.10	±0.52	±0.04	±0.39	±0.10	±0.09	±2.63	±0.14	
#174	51.95	0.89	4.64	0.58	3.87	1.02	22.42	4.9	1.13	4.16	1.95	97.5
N=2	±1.05	±0.68	±0.76	±0.05	±0.23	±0.10	±1.14	±0.76	±0.17	±0.97	±1.02	
#104	50.66	0.07	3.34	0.18	4.33	0.63	20.8	5	4.2	3.36	0.97	93.54
N=2	±0.8	±0.05	±0.01	±0.02	±0.1	±0.20	±0.30	±0.02	±0.09	±0.25	±0.29	

N : Number of analyses.

Supplementary Table 2: Experimental conditions.

	P (GPa)	T (°C)	Time (min)	fO_2 (relative to IW)	Staring composition	Phase proportions (wt%)
#1223	5	1380	120	-3.6	EC w/oS	melt (7), opx (56), coes (4), met (33)
#1216	10	1650	90	-1.8	EC w/oS	melt (1), opx (40), grt (28), st(1), met (30)
#174	20	1800	90	-2.2	EC w/oS	melt (5), maj (34), wads (11), Ca-maj (6), K-rich phase (1), met (33)
#104	25	1900	60	-2.1	EC w/S	melt (1), bridg (47), st (9), K-holl (1), met (32)

Oxygen fugacity is calculated relative to iron-wüstite buffer as in ref 5. The starting materials have an EC-like composition without (W/oS) or with (W/S) addition of sulfur (see Supplementary Table1).
opx=orthopyroxene, coes=coesite, met=metal, grt=garnet, st=stishovite, maj=majorite, wads=wadsleyite, ca-maj=Ca-majorite, bridg=bridgmanite (Mg-perovskite), K-holl=K-hollandite.

Supplementary Note 1: Chronological constraints of collisional loss on protoplanetary bodies and interaction with nebular material

The ages measured on angrites and CR chondrites indicate that the formation of chondrules could have occurred during the growth of protoplanetary bodies. Indeed, in CR chondrites, chondrules have absolute ages of 4.5647 Ga[9]. The basaltic meteorites (angrites) display Pb isotopic ages of 4.5662 Ga[10], which indicate that angrites parent body accreted and differentiated prior to the formation of chondrules in CR chondrites, showing that planetesimals and chondrules were at least contemporary. A recent study based on the determination of the initial $^{26}Al/^{27}Al$ ratios in angrites confirmed the early differentiation of angrites asteroid body that have likely accreted ~0.25 ±0.15 My after the formation of the CAI[11]. On the other hand, the formation of a vapour-melt plume through catastrophic collisions between planetary bodies was also previously proposed as being the origin of chondrules in CB carbonaceous chondrites that formed at 4.5677-4.5678 Ga[12,13].

Supplementary Note 2: The contrasting effect of collisional erosion in carbonaceous and non-carbonaceous chondrites

Collisional erosion could also explain why EH-chondrites and non-carbonaceous chondrites are less depleted in lithophile volatile elements than the oxidized carbonaceous chondrites, while both are depleted in non-lithophile volatile elements[13] (see Fig. 2 in ref [13]). When focusing on the volatility spectra of the bulk chondrites, we observe a different behaviour of the moderately volatile elements. In all chondrites, the volatile siderophile elements are depleted relative to CI, and the depletion is correlated with the degree of volatility of the elements. This is in agreement with a fractional condensation of the solids in the solar nebula.

However, the carbonaceous chondrites are the only chondrites that describe a depletion for the volatile lithophile elements. This can be interpreted as additional evidence of the collisional erosion, since only the elements abundant at the surface of the planetary embryos are volatilized during the impacts. The fact that carbonaceous chondrites are not affected by lithophile volatile enrichment might be due to their oxidized nature, which could favour more siderophile elements in the mantle. A differentiated crust formed under oxidized conditions would concentrate all incompatible elements, including a larger amount of siderophile elements compared to the case for more reduced planetary bodies. In addition, the density of the silicate melts produced under oxidized conditions should be higher, due to higher FeO-content. This could prevent an efficient ascent of melts to the surface of the planetary body[14].

Hence, there is no reason to expect a significant difference between the behaviour of the lithophile and siderophile elements for chondritic material formed close to oxidized planetary bodies (as carbonaceous chondrites).

This redox-related explanation is challenged by the observation of the enrichment in silica and alkali elements at the edges of the chondrules of carbonaceous chondrites. This could be due to the formation of their chondrules under more reducing conditions than the matrices. If so, the specific chemical signature of carbonaceous chondrites could be unravelled by the more important proportion of matrix than chondrules in carbonaceous chondrites (matrix>30%) in comparison to enstatite and ordinary chondrites (<10%)[13].

Supplementary References

1 Javoy, M. *et al.* The chemical composition of the Earth : Enstatite chondrite models. *Earth and Planetary Science Letters* **293**, 259-268 (2010).

2 Allègre, J. A., Poirier, J. P., Humler, E. & Hofmann, A. W. The chemical composition of the Earth. *Earth and Planetary Science Letters* **134**, 515-526 (1995).

3 Gessmann, C. K., Wood, B. J., Rubie, D. C. & Kilburn, M. R. Solubility of silicon in liquid metal at high pressure: implications for the composition of the Earth's core. *Earth and Planetary Science Letters* **184**, 367-376 (2001).

4 McDonough, W. F. & Sun, S. S. The composition of the Earth. *Chemical Geology* **120**, 223-253 (1995).

5 Jagoutz, E. *et al.* in *Proceedings of the 10th Lunar and Planetary Science Conference* 2031-2050 (1979).

6 O'Neill, H. S. C. & Palme, H. *Composition of the silicate Earth: implications for accretion and core formation.*, Vol. The Ringwood Volume (1998).

7 Ringwood, A. E. *Origin of the Earth and Moon.* (Springer, 1979).

8 McCoy, T., Dickinson, T. L. & Lofgren, G. Partial melting of the Indarch (EH4) meteorite: A textural, chemical, and phase relations view of melting and melt migration. *Meteoritics & Planetary Science* **34**, 735-746 (1999).

9 Amelin, Y., Krot, A. N., Hutcheon, I. D. & Ulyanov, A. A. Lead isotopic ages of chondrules and calcium-aluminum-rich inclusions. *Science* **297**, 1678-1683 (2002).

10 Baker, J. A., Bizzarro, M., N., W., Connelly, J. & Haak, H. Early planetesimal melting from an age of 4.5662 Gyr for differentiated meteorites. *Nature* **436**, 1127-1131 (2005).

11 Schiller, M., Connely, J. N., Glad, A. C., Mikouchi, T. & Bizzarro, M. Early accretion of protoplanets inferred from a reduced inner solar system ^{26}Al inventory. *Earth & Planetary Science Letters* **420**, 45-54 (2015).

12 Krot, A. N., Amelin, Y., Cassen, P. & Meibom, A. Young chondrules in CB chondrites from a giant impact in the early Solar System. *Nature* **436**, 989-992 (2005).

13 Krot, A. N., Keil, K., Goodrich, C. A., Scott, E. R. D. & Weisberg, M. K. in *Treatise on Geochemistry* Vol. 1 (ed A. M. Davis) (Elsevier, 2003).

14 Fu, R. R. & Elkins-Tanton, L. T. The fate of magmas in planetesimals and the retention of primitive chondritic crusts. *Earth and Planetary Science Letters* **390**, 128-137 (2014).
